# Perinatal Obesity Sensitizes for Premature Kidney Aging Signaling

**DOI:** 10.3390/ijms24032508

**Published:** 2023-01-28

**Authors:** Jaco Selle, Katrin Bohl, Katja Höpker, Rebecca Wilke, Katharina Dinger, Philipp Kasper, Bastian Abend, Bernhard Schermer, Roman-Ulrich Müller, Christine Kurschat, Kai-Dietrich Nüsken, Eva Nüsken, David Meyer, Soni Savai Pullamsetti, Björn Schumacher, Jörg Dötsch, Miguel A. Alejandre Alcazar

**Affiliations:** 1 Translational Experimental Pediatrics—Experimental Pulmonology, Department of Pediatric and Adolescent Medicine, University Hospital Cologne, 50931 Cologne, Germany; 2 Department of Medicine II, Nephrology Research Laboratory, University Hospital of Cologne, Faculty of Medicine, University of Cologne, 50931 Cologne, Germany; 3 Cologne Excellence Cluster on Cellular Stress Responses in Aging-Associated Diseases (CECAD), Faculty of Medicine, University of Cologne, 50931 Cologne, Germany; 4 Department of Pediatric and Adolescent Medicine, University Hospital Cologne, 50931 Cologne, Germany; 5 Department of Gastroenterology and Hepatology, University Hospital Cologne, Faculty of Medicine, University of Cologne, 50931 Cologne, Germany; 6 Center for Molecular Medicine Cologne (CMMC), University Hospital Cologne, Faculty of Medicine, University of Cologne, 50931 Cologne, Germany; 7 Institute for Genome Stability in Aging and Disease, Medical Faculty, University Hospital Cologne, Faculty of Medicine, University of Cologne, 50931 Cologne, Germany; 8 Department of Lung Development and Remodeling, Max-Planck-Institute for Heart and Lung Research, Member of the German Center for Lung Research (DZL), 61231 Bad Nauheim, Germany; 9 Institute for Lung Health (ILH), Universities of Gießen and Marburg Lung Centre (UGMLC), Cardiopulmonary Institute (CPI), Member of the German Center of Lung Research (DZL), 35392 Gießen, Germany

**Keywords:** chronic kidney disease, perinatal obesity, DNA damage response, kidney aging

## Abstract

Chronic Kidney Disease (CKD), a global health burden, is strongly associated with age-related renal function decline, hypertension, and diabetes, which are all frequent consequences of obesity. Despite extensive studies, the mechanisms determining susceptibility to CKD remain insufficiently understood. Clinical evidence together with prior studies from our group showed that perinatal metabolic disorders after intrauterine growth restriction or maternal obesity adversely affect kidney structure and function throughout life. Since obesity and aging processes converge in similar pathways we tested if perinatal obesity caused by high-fat diet (HFD)-fed dams sensitizes aging-associated mechanisms in kidneys of newborn mice. The results showed a marked increase of γH2AX-positive cells with elevated 8-Oxo-dG (RNA/DNA damage), both indicative of DNA damage response and oxidative stress. Using unbiased comprehensive transcriptomics we identified compartment-specific differentially-regulated signaling pathways in kidneys after perinatal obesity. Comparison of these data to transcriptomic data of naturally aged kidneys and prematurely aged kidneys of genetic modified mice with a hypomorphic allele of *Ercc1*, revealed similar signatures, e.g., inflammatory signaling. In a biochemical approach we validated pathways of *inflammaging* in the kidneys after perinatal obesity. Collectively, our initial findings demonstrate premature aging-associated processes as a consequence of perinatal obesity that could determine the susceptibility for CKD early in life.

## 1. Introduction

Chronic kidney disease (CKD) is a global socioeconomic health burden with a rising prevalence over the last decade, leading to increasing numbers of patients with kidney failure. While the exact cause for the increase of kidney failure remains unclear, CKD is strongly associated with age-related kidney function decline, hypertension, and diabetes [1]. The continuous loss of renal glomerular and tubular function with age [2,3,4] is associated with both macroscopic and microscopic changes, e.g., a reduction in renal size, an increase in adipose tissue and extracellular matrix, a higher frequency of glomerulosclerosis [5,6,7,8,9], and a decrease in the number of nephrons in elderly persons when compared to those under 30 years of age [10,11]. This age-related impairment of kidney structure and function is of great clinical importance and has imminent impact on the susceptibility to maladaptive repair mechanisms and finally CKD.

Aging processes can be affected by various endogenous and environmental factors, including obesity. Epidemiological studies demonstrate that obesity has implications on cardiovascular diseases (CVD) as well as CKD [12]. For instance, various studies reported an association between obesity and kidney diseases, e.g., CKD and propose high body mass index (BMI) as one of the strongest risk factors for a new-onset of CKD [13,14,15]. Moreover, Silverwood and colleagues highlighted the impact of onset and duration of obesity on CKD and show that becoming overweight at age 26 to 36 years increases the risk of developing CKD when compared with those who first became overweight at age 60–64 years, or never became overweight [16]. These results strongly imply that the time window of exposure to obesity and associated metabolic disorders is critical in determining the risk for CKD.

Obesity and being overweight are a pandemic and a growing socioeconomic burden. In Europe approximately 50% of the adult population is overweight and 20% is obese [17,18,19], affecting also women of child-bearing age. Consequently, these rising numbers of obese women lead to an exposure of the fetus or the newborn to an impaired maternal metabolism, e.g., increased adipocytokines or hyperinsulinemia. The concept of early origins of diseases comprises the idea that adverse influences during organ development lead to long-term programming of physiology, structure, and function of an organ [20,21,22]. Maternal overweight and obesity were identified as a predisposing factor for CKD in young children and in adulthood [23,24]. Similarly, prior animal studies of our group demonstrated that maternal obesity has long-lasting effects on the offspring’s health [25,26], including compartment-specific structural and functional changes similar to early characteristics of CKD. However, the molecular mechanisms by which maternal high-fat diet (HFD) and obesity program kidney development and increases the risk for functional impairment remain uncertain.

Aging-related processes are intimately linked to the activation of cellular stress responses defining hallmarks of aging, including increased reactive oxygen species (ROS) and altered antioxidant capacity, higher DNA damage response (DDR) with genomic instability, changes in cell cycle progression and senescence, inflammatory response as well as dysregulation of growth factor and nutrient sensing signaling [27,28,29,30,31]. Obesity is linked to hyperactive adipose tissue that secretes inflammatory adipocytokines, e.g., leptin and interleukin-6 (IL-6), which cause, with prolonged exposure, subacute chronic inflammation and insulin resistance. Both inflammation and insulin resistance are early characteristics of obesity as well as of CKD, being apparent when the GFR is still unaffected [32,33,34]. Interestingly, dysfunction of the adipose tissue has been identified as an important hallmark of the aging process through a systemic pro-inflammatory state (*inflammaging*) and insulin signaling [32,35]. Since CKD is an age-associated disease and obesity shares similar processes with aging, it is likely that these conditions converge in common cellular pathways.

**Figure 1:**
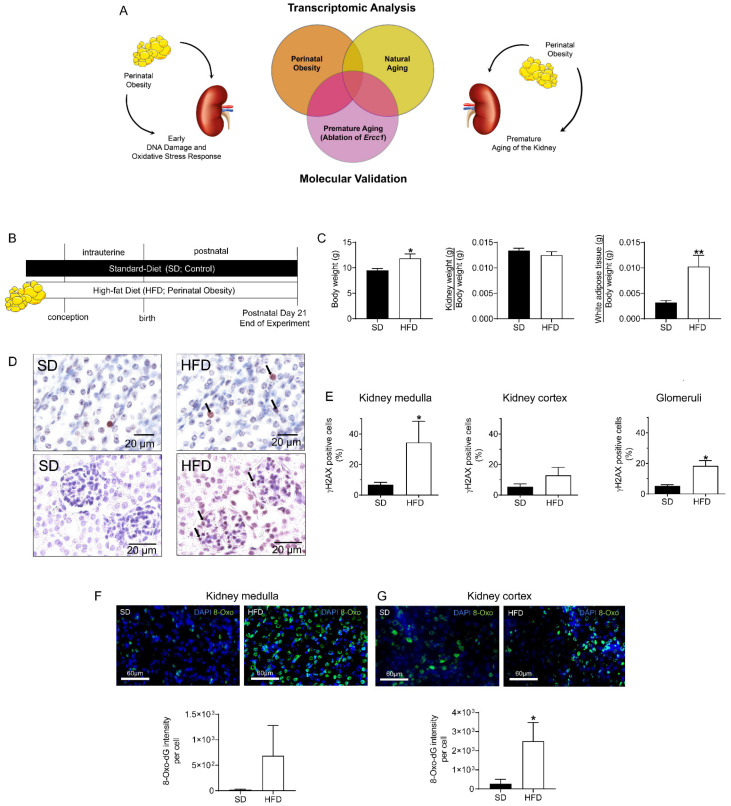
(**A**): Study design: Perinatal obesity induces aging-associated processes, e.g., DNA damage response, and causes premature aging of the kidneys with increased susceptibility for chronic kidney diseases later in life. Identification of converging signaling pathways using transcriptomics of kidneys from male offspring at postnatal day 21 (P21) after perinatal obesity, naturally-aged kidneys and prematurely-aged kidneys of genetic modified mice with an ablation or *Ercc1*. (**B**): Scheme illustrating the experimental mouse model of perinatal obesity, in which male offspring of high-fat diet (HFD, perinatal obesity) or standard diet (SD, control) fed dams are investigated at P21. (**C**): Body weight (g), kidney weight (g) to body weight (g) ratio, and white adipose tissue (g) to body weight (g) ratio in male offspring at P21. (**D**,**E**): Assessment of DNA damage response (DDR) using γH2AX as an indicator in kidneys at P21. Representative immunohistochemical γH2AX staining of cortical and medullary compartments (**D**); black arrows indicate γH2AX positive nuclei. Percentage of γH2AX positive cells related to all cells is shown for medulla, cortex, and glomeruli (**E**). (**F**,**G**): Immunofluorescence staining for 8-Oxo-dG (RNA/DNA damage) in kidney medulla and cortex as an indicator of oxidative stress in kidneys at P21. Relative 8-Oxo-dG intensity per cell is indicated in the graph under the respective images. *n* = 5–6 per group; Mean ± SEM; Unpaired or Mann-Whitney test; * *p* < 0.05, ** *p* < 0.01.

Here, we aimed to investigate if perinatal obesity induces a premature aging profile of the kidney using comprehensive transcriptomic analysis of three groups: perinatal obesity, natural aging, and premature aging (ablation of *Ercc1*) (Figure 1A). We identified compartment-specific differentially regulated signaling pathways in kidneys after perinatal obesity using unbiased comprehensive transcriptomics. Furthermore, the comparison of these data to transcriptomic data of naturally aged kidneys and prematurely aged kidneys of genetic modified mice with a hypomorphic allele of *Ercc1*, revealed similar signatures, e.g., inflammatory signaling, indicating that kidney mechanisms after maternal obesity convey the hallmarks of aging.

## 2. Results

### 2.1. Maternal HFD Induces DNA Damage Response (DDR) and Oxidative Stress in Male Offspring’s Kidney at P21

First, we tested the hypothesis that perinatal obesity activates aging processes such as DNA damage response (DDR) and oxidative stress response. Perinatal obesity was induced by maternal HFD before conception as well as during gestation and lactation. At P21, the male offspring of HFD-fed dams showed increased body weight, no differences in relative kidney weight, but an elevated relative white adipose tissue (WAT) when compared to offspring of SD-fed dams (Figure 1B,C). To determine if perinatal obesity induces the DDR and oxidative stress in kidneys early in life as an indicator of premature aging, we stained kidney slides for γH2AX and 8-Oxo-dG (8-Oxo-7,8-dihydro-2′-deoxyguanosine) at P21, respectively. 8-Oxo-dG serves as a marker for DDR produced by oxidants. We separated medulla (predominately collecting duct epithelia) and kidney cortex (predominantly glomeruli and tubuli) in order to appreciate the fundamental differences in the biology of these two kidney regions. We observed a four-fold increase of γH2AX^+^ nuclei in the kidney medulla of mice exposed to perinatal obesity. Assessment of DDR in the kidney cortex showed a similar, but not significant, trend. Interestingly, exclusive analysis of the glomeruli showed a four-fold increase in γH2AX^+^ nuclei (Figure 1D,E). We then analyzed the intensity of 8-Oxo-dG per cell in kidneys at P21 and found an increase in medullar cells after perinatal obesity. In addition, we detected a marked elevated amount of 8-Oxo-dG per cell in the cortical compartment (Figure 1F,G). Collectively, these findings indicate increased DNA damage in cells of the medulla and glomeruli in kidneys of male offspring of HFD-fed obese dams.

### 2.2. Perinatal Obesity Induces Aging-Associated Pathways

**Figure 2:**
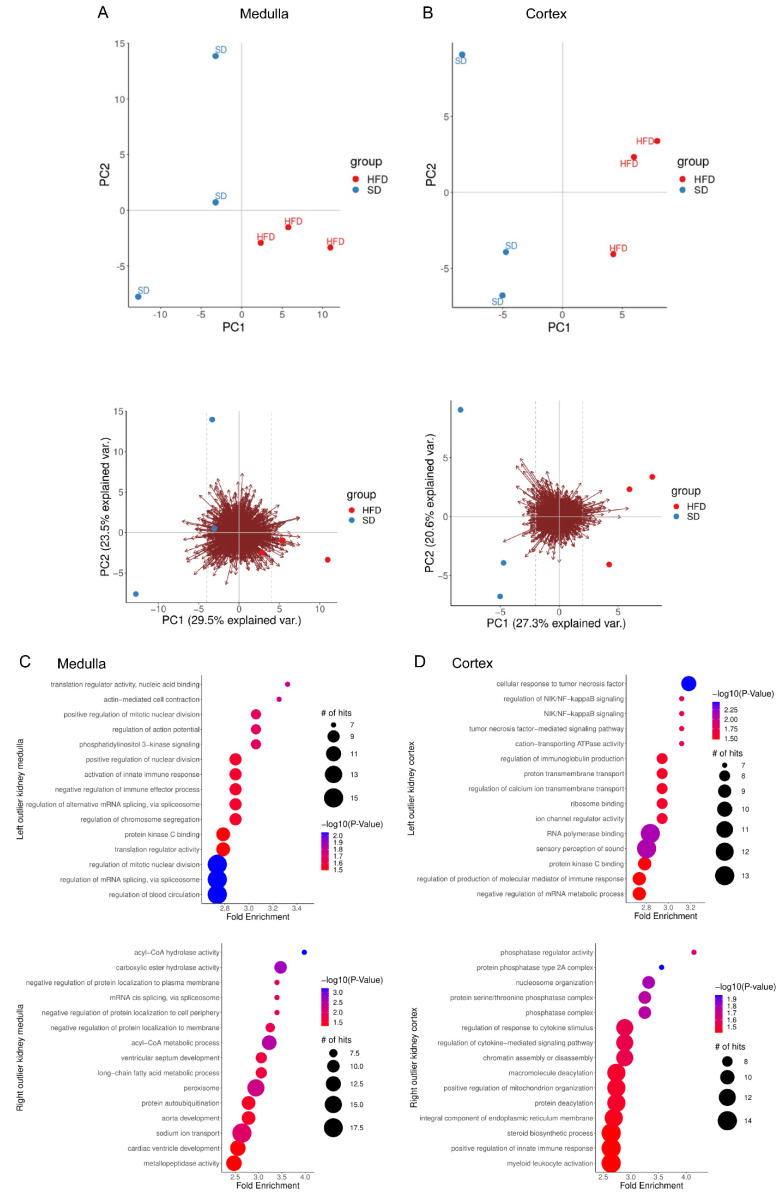
Comparison of kidney’s transcriptome from male offspring with maternal high-fat diet (HFD) or standard diet (SD). Cortex and medulla were studied separately. (**A**,**B**): Medulla and cortex, respectively: PCA of all genes. The lower plots show the biplots: the PCA scores together with the loadings (arrows) of all genes. The dashed lines indicate the cutoffs used to define left and right outliers. (**C**,**D**): Medulla and cortex, respectively: Bubble plots of top 15 enriched GO-terms of left and right outliers shown in upper and lower plot, respectively. *p* value depicted as color code, number of annotated genes corresponding to bubble size.

The preceding findings demonstrate that perinatal obesity induces key hallmarks of aging early in life. We next chose a non-biased transcriptomic experiment to examine kidney cortex and kidney medulla of our experimental mice separately using RNA-Seq. Principal component analyses show clustering of the distinct organ samples of the male offspring of obese or control dams (Figure 2A,B). Subsequently, we studied the differentially regulated signaling pathways in kidneys between male offspring with perinatal obesity (HFD) and control (SD). To this end, separate analysis of differentially regulated GO-term signaling pathways for kidney cortex and medulla were performed. The data demonstrated a compartment-specific transcriptomic signature in kidney medulla and cortex induced by perinatal obesity. In kidney medulla, we found metabolic processes (e.g., Acyl-Co metabolic processes, peroxisomes, long-chain fatty acid metabolic processes), cell division, PIK3 signaling (upstream of AKT signaling), and RNA biology processes differentially regulated. In contrast, in the cortex, we identified inflammatory signaling and immune response (e.g., NFκB or TNFα signaling) as well as pathways involved in DNA homeostasis (e.g., chromatin assembly and transcriptional regulation), and posttranslational modifications (e.g., phosphatases) (Figure 2C,D).

### 2.3. Perinatal Obesity Induces Compartment-Specific Activation of Inflammatory STAT3 and NFκB Signaling Cascade As Well As A Dysregulation of the Aging-Associated Renal IGF1R/AKT Pathway

**Figure 3:**
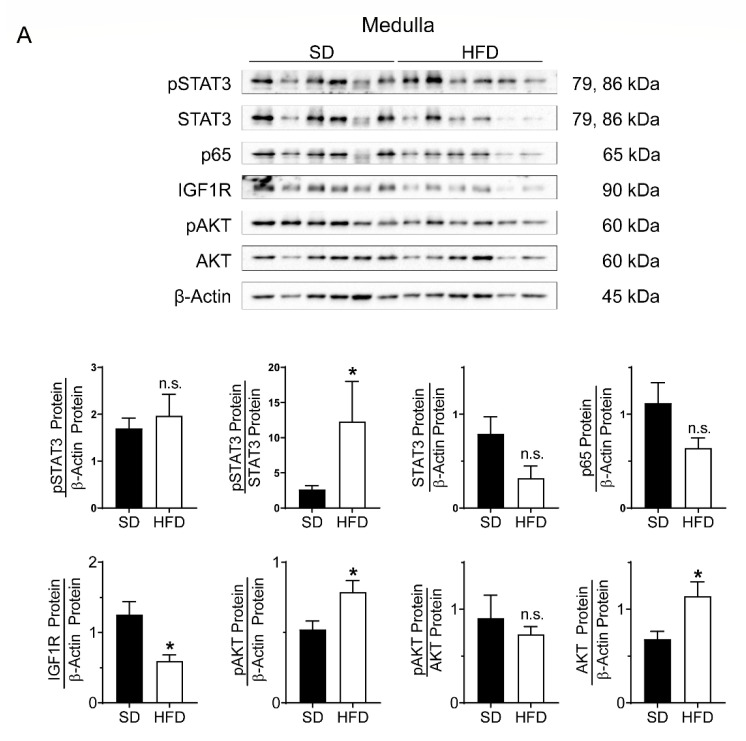

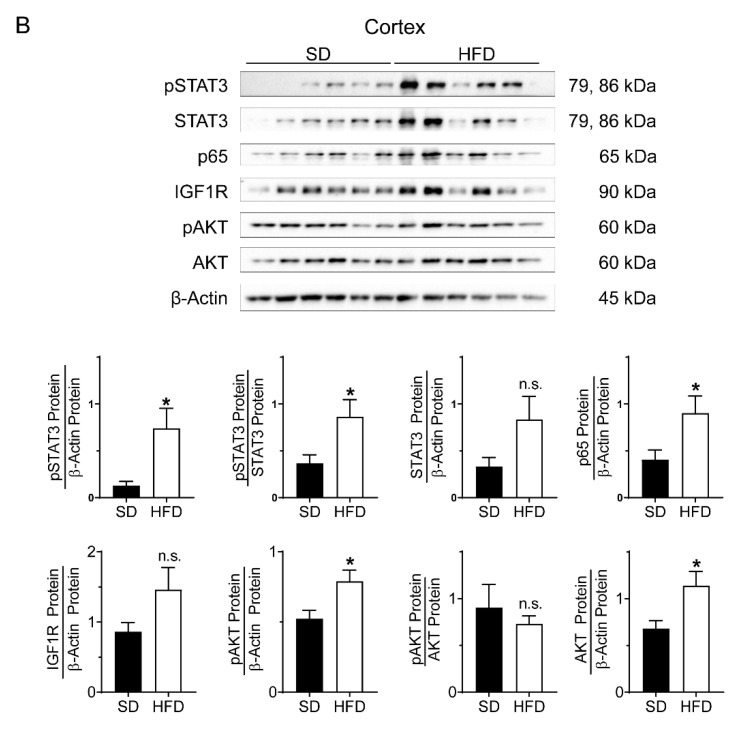
(**A**,**B**): Assessment of STAT3 signaling, p65 protein abundance, insulin-like growth factor 1 receptor (IGF1-R) abundance, and AKT signaling using total homogenates of kidney medulla (**A**) and cortex (**B**) from male offspring at postnatal day 21 (P21) after perinatal obesity: Immunoblots show phosphorylated STAT3 (pSTAT3) and total STAT3; p65 as an indicator of NFκB signaling; IGF1R protein as well as phosphorylated AKT (pAKT) and total AKT; β-Actin served as loading control. Quantitative densitometric analyses are shown below the immunoblots: quantitative summary displays pSTAT3 relative to total STAT3 or to β-Actin as well as total STAT3 relative to β-Actin; quantification of p65 relative to β-Actin; IGF1R was related to the loading control β-Actin; quantitative summary displays pAKT relative to total AKT or to β-Actin as well as total AKT relative to β-Actin. Standard diet (SD), high-fat diet (HFD, perinatal obesity); *n* = 6 per group; Mean ± SEM; Mann-Whitney; * *p* < 0.05; n.s. = not significant.

To confirm inflammation as another hallmark of aging in kidneys after perinatal obesity, we performed compartment-specific immunoblots for the STAT3 signaling pathway and for p65 as an indicator of the NFκB signaling cascade (Figure 3A,B). In the kidney medulla, we found a higher amount of phosphorylated STAT3 (pSTAT3) relative to total STAT3 after perinatal obesity than control, whereas p65 was similar in both groups. In the kidney cortex, we determined an activation of pSTAT3 relative to total STAT3 and relative to β-Actin after perinatal obesity. This marked inflammatory response in the kidney cortex was supported with a 2-fold increase of p65, indicative of an activated NFκB signaling cascade. In the kidney medulla the increased DDR and oxidative stress response are related to a reduction of IGF1R by 50%, whereas phosphorylated AKT (pAKT) relative to the loading control is increased after perinatal obesity (Figure 3A,B). Activation of AKT signaling could be mediated by hyperinsulinemia that has been described in our prior studies [26]. Collectively, we provide evidence of dysregulation of aging-associated renal IGF1R/AKT after perinatal obesity that might be related to hyperinsulinemia, supporting the notion of premature aging processes.

### 2.4. Converging Aging Signaling in Kidney after Perinatal Obesity

**Figure 4:**
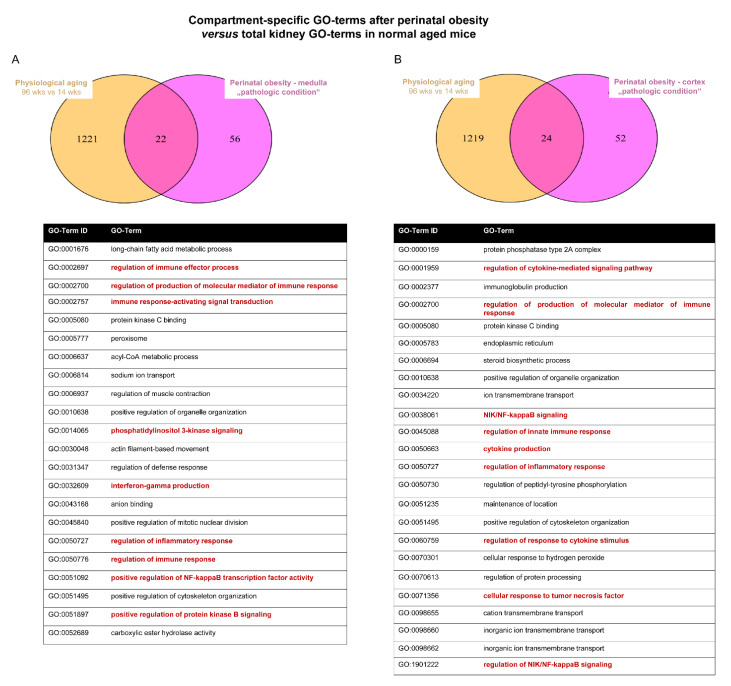

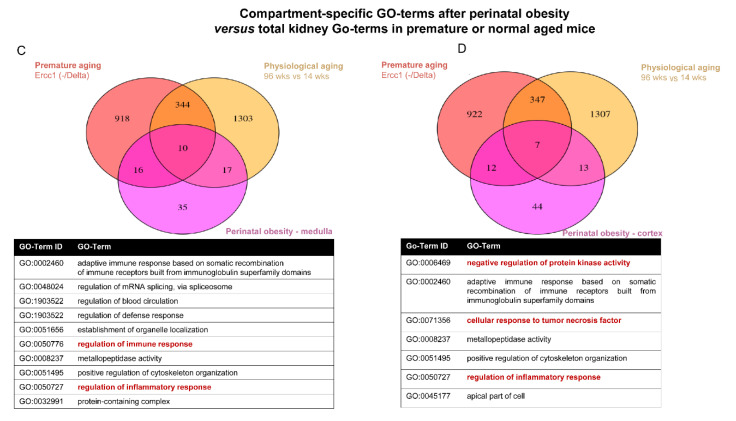
(**A**,**B**): Venn diagrams of significant (*p* < 0.05) GO-terms show overlaps of naturally aged total kidneys and kidney medulla (**A**) or cortex (**B**) after perinatal obesity. (**C**,**D**): Venn diagrams of significant (*p* < 0.05) GO-terms show overlaps in the three conditions: prematurely aged (total kidney), naturally aged (total kidney) and perinatal obesity (medulla, (**C**); cortex, (**D**)). Tables show the terms shared among all two (**A**,**B**) or three (**C**,**D**) conditions.

Next, we tested if perinatal obesity causes premature renal aging signaling. To this end, we first used transcriptomic data from kidneys of naturally aged mice at 96 weeks of age [36]. In both medulla and cortex, the comparison of the GO-terms showed that about 30% of the GO term identified in the SD/HFD transcriptomic overlapped with those of physiologically aged kidneys (medulla: 22/78; cortex: 24/76; Figure 4A,B). Importantly, these terms contain inflammation-related pathways, e.g., “NFκB transcription factor activity,” and “immune response activating transcription induction” or “phosphatidylinositol 3-kinase signaling.” Second, we used transcriptomic data from prematurely aged kidneys of genetic modified mice with an ablation of *Ercc1*, a regulator of DNA repair; loss of Ercc1 causes an accumulation of DNA damage and results in premature aging [37]. Approximately 30% of the GO-terms of prematurely aged kidneys overlapped with naturally aged kidneys (354/1288). Similarly, the comparison of GO-terms of kidney after perinatal obesity with those of prematurely-aged and naturally-aged kidney showed an overlapping rate of about 30% [medulla: 26/78 (prematurely aged), 27/78 (naturally aged); cortex: 19/76 (prematurely aged), 20/76 (naturally aged)] (Figure 4C,D). Overlap of the differential regulated pathways of these three experimental systems does not only demonstrate that there is a compartment-specific marked number of converging pathways between perinatal obesity and aging, but also highlights key hallmarks, notable amongst those inflammatory response (e.g., regulation of cytokine-mediated signaling pathway), regulation of DNA and RNA biology (e.g., regulation of mRNA splicing, via spliceosome), and organelle function (e.g., positive regulation of organelle organization).

## 3. Methods

### 3.1. Animal Procedures

All animal procedures were performed in accordance with German regulations and legal requirements and were approved by the local government authorities (LANUV, NRW, Germany; AZ # 2012A424; 2018.A230). We used virgin female mice (C57Bl/6N) from our own colony as future dams. Mice were housed in a room at 22 ± 2 °C and exposed to a 12-h dark/light cycle. To induce maternal obesity female mice received a high-fat diet (HFD^mat^; modified #C1057, Altromin, Lage, Germany) for 8–10 weeks after weaning [postnatal day 21 (P21)]. Control dams were fed standard diet (SD, Control^mat^; ssniff #R/M-H, V1534-0). Both HFD^mat^ and Control^mat^ dams were mated with SD-fed males and continued on their respective diet throughout pregnancy and lactation. After birth, the litter size of all dams was normalized to six for each litter. Water and chow were available *ad libitum*. Exact numbers of animals are listed in the figure legends. In the present study, we focused on male offspring for two reasons: first, the effect of maternal HFD differs between male and female offspring [38,39]; second, prior studies from our group investigated the impact of perinatal obesity or HFD on lung development and early origins of chronic lung disease only in male mice [40,41,42,43]. Therefore, we only included male mice in the present experiments; however, future studies should address sex-specific differences of perinatal obesity in kidneys. Data from Ercc1^-/Δ^ mice were reported and described previously [37].

### 3.2. Physiological Data of Dams and Offspring

Male offspring body and renal (right kidney) weights were obtained at P21.

### 3.3. Tissue Preparation

Mice were sacrificed and the kidneys were excised at P21. While the right kidney was immediately separated into medulla and cortex, and frozen in liquid nitrogen, the left kidney was fixed in 4% paraformaldehyde in phosphate-buffered saline (PBS) for histology preparation.

### 3.4. RNA Sequencing and Transcriptomic Data Analysis

Total RNA was isolated from renal tissue as described before [26]. Subsequently, RNA sequencing (RNA-Seq) was performed. The reads were trimmed of single-end Illumina adapters and regions of low quality with Trimmomatic (version 0.36) [44]. The trimmed reads were mapped to the GRCm39 mouse reference genome with STAR version 2.6.0 [45] using default parameters. The analysis of differential gene expression was performed using the R package DeSeq2 (version 1.26.0) [46,47].

A standard principal component analysis (PCA) was done for all genes with a normalized read count of at least 10 using the R function prcomp. The PCA was calculated with a matrix of fold changes (each measurement versus the mean over all samples) instead of the counts. The loadings of the first (PC1) and second principal component (PC2) were visualized in a scatter plot. Most interesting are the genes with the most extreme values on the first principal component which explains approximately 28% and 30% of variance for cortex and medulla, respectively. The second principal component also explains a big proportion (approximately 21% and 24% for cortex and medulla, respectively) of the variance in the data. We investigated which factor contributes most likely to this principal component (e.g., different litters, cages) but we could not find a known factor and therefore focused on the first principal component.

Genes with a high positive value in PC1 are “HFD related” and with a high negative value in PC1 are “SD related.” We defined lower (“SD related”) and upper (“HFD related”) outliers based on a cutoff of the loadings of the genes of +/− 0.04, i.e., defining the outer 5.5% or 5.9% of genes as outliers for medulla and cortex, respectively. For these “outlier” gene sets, gene ontology enrichments and gene set enrichment analysis were calculated. GO enrichment was performed with the R package topGO. Gene set enrichment analysis was performed with the R package clusterProfiler (version 3.14.3) using the method gseGO [48,49].

To test the hypothesis that perinatal obesity causes premature renal aging as a result of activation of signaling pathways that converge with aging processes, we compared GO enrichment analyses of medulla and cortex separately with GO enrichment analyses of two external transcriptomic datasets: (1) transcriptomic data from kidneys of naturally-aged mice at 96 weeks of age [36], and (2) transcriptomic data from prematurely-aged kidneys of genetically modified mice with an ablation of *Ercc1* which leads to premature aging [37]. We report these four comparisons separately for medulla and cortex with Venn diagrams.

### 3.5. Immunoblots

The immunoblots were performed on protein isolations of kidney medulla and kidney cortex homogenates as previously described [26]. Blots were incubated with the following antibodies: rabbit anti-mouse pAKT (#4058, 1:1000, Cell Signaling, Danvers, MA, USA), rabbit anti-mouse AKT (#9272, 1:2000, Cell Signaling, Danvers, MA, USA), rabbit anti-mouse Insulin-like growth factor receptor 1 (IGF1R) (#3027, 1:1000, Cell Signaling, Danvers, MA, USA), rabbit anti-mouse pSTAT3 (#9145, 1:1000, Cell Signaling, Danvers, MA, USA), mouse anti-mouse STAT3 (#9139, 1:3000, Cell Signaling, Danvers, MA, USA), rabbit anti-mouse p65 (#8242, 1:1000, Cell Signaling, Danvers, MA, USA), and with mouse anti-mouse β-Actin (#3700, 1:10.000, Cell Signaling, Danvers, MA, USA) serving as the loading control. Anti-mouse IgG, HRP-linked (#7076, Cell Signaling, Danvers, MA, USA) and anti-rabbit IgG, HRP-linked (#7074, Cell Signaling, Danvers, MA, USA) were used as secondary antibodies. Total protein was detected in each case after removal of the respective phosphorylated protein using β-mercaptoethanol on the same immunoblot.

The original immunoblots are available DOI: 10.6084/m9.figshare.20489799.

### 3.6. Immunohistochemistry

PFA-fixed and paraffin-embedded kidney tissue sections (3 µm) were deparaffinized in Neoclear (Sigma-Aldrich, #109843, St. Louis, MO, USA) and rehydrated in a graded ethanol series (96%, 70% and 30% for 5 min each) to H_2_O. Antigen retrieval was performed with Rodent Decloacer (Zytomed Systems GmbH, RD913L, Berlin, Germany) at 90–120 °C for 20 min. Peroxidase block was performed in 3% H_2_O_2_ for 10 min at room temperature. Afterwards, tissue was blocked (Sea Block, Thermo Scientific™, #37527, Waltham, MA, USA) at room temperature for 1 h. Primary antibody rabbit anti-mouse γH2AX (S139, γH2AX; Cell signaling, #2577, 1:1000, Danvers, MA, USA) was applied overnight at 4 °C. Following primary antibody incubations tissues section were treated with ZytoChem Plus HRP Polymer (Zytomed Systems GmbH, ZUC053, Berlin, Germany) for 30 min at room temperature. Tissue was then incubated in AEC permanent (Zytomed Systems GmbH, ZUC054, Berlin, Germany) for 5 min and subsequently counterstained with hematoxylin. Analysis was performed by light microscopy using 10 random fields of view per kidney section, for medulla and cortex separately. Quantification of γH2AX staining was performed by counting the percentage of γH2AX-positive nuclei relative to all nuclei per field of view.

### 3.7. Immunofluorescent Staining

PFA-fixed and paraffin-embedded kidney tissue sections (3 µm) were deparaffinized in Neoclear (Sigma-Aldrich, #109843, St. Louis, MO, USA) and rehydrated in a graded ethanol series (100%, 96%, 80% and 70% for one minute each) to PBS. The tissue was incubated with solution A from the MaxBlock Autofluorescence Reducing kit (MaxVision Biosciences, #MB-L, Bothell, WA, USA) according to the manufacturer’s instructions and washed with 60% EtOH and PBS. Antigen retrieval was performed with 10 mM citrate buffer pH6 (Dako, Cat. No. S2369, Santa Clara, CA, USA) at 90–120 °C for 25 min. Sections were blocked with Sea Block (Thermo Fisher Scientific, #37527, Waltham, MA, USA), and incubated at 4 °C overnight with mouse anti-mouse DNA/RNA damage antibody (8-Oxo-dG; Abcam, ab62623, 1:400, Cambridge, UK). Subsequently, slides were incubated with the respective goat-raised secondary antibody and conjugated with an F488 fluorochrome (Jackson Laboratory, #115-485-003, 1:1000, Bar Harbor, ME, USA) for 1 h at room temperature. Cell nuclei were stained with DAPI (#D9542, Sigma-Aldrich, St. Louis, MO, USA). Tissue sections were incubated with solution B from the MaxBlock Autofluorescence Reducing kit (MaxVision Biosciences, #MB-L, Bothell, WA, USA) and mounted with glass coverslips using Fluoromount (Sigma-Aldrich, #F4680, St. Louis, MO, USA). Photomicrographs of representative images were taken using a microscope (Olympus BX43, Hamburg, Germany) and a 40× and 100× lens using CellSens Dimension software (Olympus). Quantification of 8-oxo-dG was performed by measuring 8-oxo-dG Integrated Density per field of view in correlation to cell nuclei numbers.

### 3.8. Analysis of Data

Values are shown as means ± standard error of the mean (SEM). To test for statistical difference, we performed Mann-Whitney test or Student *t*-test. A *p*-value less than 0.05 was considered statistically significant.

## 4. Discussion

Obesity is linked to hyperactive adipose tissue with an inflammatory secretory phenotype including leptin and IL-6 that cause, when prolonged, subacute chronic inflammation and insulin resistance. An increasing number of studies reported the crucial role of obesity and obesity-induced inflammation in the development of CKD [50]. Furthermore, a link between obesity, adipocytokines, and the progression of CKD has been identified [51]. Not only the diet and caloric intake itself, but also the composition of the diet plays a crucial role for kidney diseases [52]. Conversely, it has been shown that caloric restriction reduces inflammation and protects against acute kidney injury [53]. However, a weight loss induced by a ketonic-diet, which is characterized by low-carbohydrate intake and animal-based diet, may lead to an increased risk for CKD [52]. Furthermore, an altered lipid profile can be seen in aging kidneys. Here, genes or proteins involved in the lipid metabolism are most prominently changed by aging. This goes along with an increased expression of genes for cellular and immune responses [54]. Clinical and experimental evidence indicate that malnutrition, ranging from reduced intrauterine nutritive supply with intrauterine growth restriction (IUGR) to high caloric intake and maternal obesity, adversely affects kidney development, structure, and function later in life [26,55,56,57,58]. Experimental data demonstrate that maternal HFD causes perinatal inflammation and adverse kidney development in offspring [59,60,61]. While exposure of the developing kidney to perinatal obesity induces kidney injury and disease, the impact of perinatal obesity on compartment-specific premature cortical and medullary aging of the kidney has yet not been addressed.

Dysfunction of the adipose tissue has been identified to contribute to a systemic pro-inflammatory state and promote aging (*Inflammaging*) [35,62,63,64]. For example, senescence of adipose tissue causes an inflammatory senescence-associated secretion phenotype (SASP), thereby inducing ROS and inflammation in other organs [63,65,66]. Hence, accumulation of dysfunctional adipose tissue could favor aging processes in other organs and early-onset of age-related diseases. For example, Yue Sun and colleagues showed that long-term HFD feeding causes kidney injury by inducing oxidative stress and mitochondrial dysfunction [67]. Prior studies from our group confirmed that perinatal obesity leads to dysfunctional adipose tissue early in life and a low-grade inflammatory state with higher IL-6 [25,26,42,68,69]. In the present study, we found a compartment-specific activation of both STAT3 and NFκB signaling cascade. This inflammatory response after perinatal obesity could contribute and trigger oxidative stress along with DDR in kidneys early in life. Metabolic along with growth factor dysregulation is another well-recognized trigger of accelerated aging. For example, diminished insulin-like growth factor-1 (IGF1) signaling extends lifespan, and late-life targeting of the IGF1R improves the healthspan and lifespan in female mice [70,71,72]. Interestingly, there is evidence that IGF1R and accumulation of DNA damage are linked to regulation of longevity [73]. In fact, here we show that increased DNA damage and oxidative stress-response is related to a dysregulation of aging-associated renal IGF1R/AKT after perinatal obesity. In fact, metabolic syndrome, including hyperinsulinemia and insulin resistance, are linked to age and could determine the risk for CKD [74,75]. Prior studies along with the present data show that perinatal obesity leads to early postnatal hyperinsulinemia, impaired glucose tolerance and renal compartment-specific activation of AKT signaling [26]. Collectively, we provide evidence of dysregulation of aging-associated renal IGF1R/AKT after perinatal obesity that might be related to hyperinsulinemia, supporting the notion of premature aging processes.

Next, we tested the hypothesis that perinatal obesity causes premature renal aging as a result of activation of signaling pathways that converge with aging processes. To this end, we used transcriptomic data from kidneys of naturally aged mice at 96 weeks of age [36] as well as from prematurely aged kidneys of genetically modified mice with an ablation of *Ercc1*, a regulator of DNA repair [37]. The comparison of GO-terms of kidneys after perinatal obesity with those of prematurely aged and naturally aged kidneys showed an overlapping rate of about 30%. Inflammatory signaling pathways play a central role in both obesity and aging. Interestingly, overlap of the differential regulated pathways of these three experimental systems identified converging pathways between perinatal obesity and aging, notable amongst those inflammatory response and regulation of DNA and RNA biology. These findings emphasize inflammation as a possible converging process along with unresolved DNA damage.

The present study identifies converging signaling pathways of metabolic and aging processes in young kidneys after perinatal obesity, indicating premature-aging in the kidney. Collectively, our results show that perinatal obesity early in life (i) leads to compartment-specific aging-associated gene signatures in kidney medulla and cortex, (ii) regulates pathways involved in inflammatory signaling (e.g., NFκB, TNFα) with compartment-specific activation of inflammatory signaling pathways (STAT3 and NFκB), post-transcriptional regulation, and DNA homeostasis, and (iii) directs signaling pathways that converge with aging processes. These findings together with prior studies of our group that demonstrate kidney matrix remodeling and a functional renal tubular decline are comprising numerous hallmarks of aging and strongly indicate that perinatal obesity sensitizes for premature kidney aging and could thereby increase susceptibility for CKD.

Some technical and experimental limitations need to be considered when interpreting the data: first, the kidney compartments analyzed in the present study using bulk RNA-Seq differ between the experimental approaches. For example, kidneys were separated into renal medulla and cortex after perinatal obesity. In contrast, transcriptome analysis in kidneys of mice with premature aging was performed on the entire kidney. Second, transcriptome analysis by bulk RNA-Seq does not provide information about cell-specific effects or changes in cell populations. Cells of different kidney compartments, e.g., the glomeruli versus distal tubules, fulfill different functions and therefore differ significantly in their expression profile. To investigate the effect of perinatal obesity on the cellular landscape of the kidney, single cell RNA-Seq should be used in future studies. Third, in the present study, we only examined the male offspring after perinatal obesity. Thus, we do not address sex-specific differences in response to perinatal obesity. Fourth, since we primarily aimed to test if perinatal obesity induces an early (premature) transcriptomic aging profile in kidneys, we only performed the analysis at P21. Accordingly, we cannot draw conclusions about the long-term effects of perinatal obesity on the renal transcriptome. To address this aspect, a later time point in adult mice after perinatal obesity is necessary.

The increasing prevalence of CKD emphasizes the emerging need of exploring new preventive and therapeutic avenues. While the pathogenesis of CKD has been extensively studied over the last decades, it remains unclear which factors determine susceptibility to CKD. In addition to genetic and epigenetic factors, there is increasing evidence that early adverse events and influences determine renal health. Aging and metabolic processes converge in similar signaling pathways. Here we show, using comprehensive system biology approaches, that perinatal obesity can induce aging-associated processes, such as inflammatory signaling, immune response, metabolic processes, and dysregulation of phosphatidylinositol 3-kinase signaling, thereby sensitizing the kidneys of male offspring to CKD. These transcriptomic data were further supported by protein analysis showing activation of inflammatory STAT3/NFκb signaling, AKT signaling, DNA damage response, and oxidative stress. These findings together with our previous study in which we demonstrated hyperinsulinemia as well as functional and structural kidney impairment in male offspring after perinatal obesity [26] indicate an activation of metabolic and inflammatory signaling possibly driving premature aging of the kidneys after perinatal obesity. Targeting the activation of aging signaling could be a novel preventive strategy for CKD.

## Data Availability

Sequencing data are available in the ArrayExpress database (https://www.ebi.ac.uk/biostudies/arrayexpress) under accession number E-MTAB-10954.
